# Bedside Clinical Ultrasound Performed by Family Physicians in Adult Patients With Abdominal Pain in a Hospital Emergency Department: Protocol for a Pilot Quasi-Experimental Study

**DOI:** 10.2196/82393

**Published:** 2026-01-02

**Authors:** Laura Carbajo Martín, Ignacio Párraga-Martínez, Luis M Beltrán-Romero, Máximo Bernabeu Wittel, Northern Huelva Health Management Area Research Group

**Affiliations:** 1 Emergency Department, Northern Huelva Health Management Area, Avda de la Esquila, Minas de Riotinto, Spain, 34 959025200; 2 Emergency and Continuous Care Group of the Spanish Society of Family and Community Medicine, Barcelona, Spain; 3 Spanish Red Cross Nursing School, University of Seville, Sevilla, Spain; 4 Health Center Zone VIII Albacete, Integrated Care Management Albacete, Albacete, Spain; 5 Preventive Medicine and Public and Community Health, Faculty of Medicine of Albacete, University of Albacete, Albacete, Spain; 6 IDISCAM, Castilla-La Mancha Institute of Health Research, Albacete, Spain; 7 Internal Medicine Department, Virgen del Rocío University Hospital, Sevilla, Spain; 8 Department of Medicine, University of Seville, Sevilla, Spain; 9 See Acknowledgments

**Keywords:** abdominal pain, emergency, personal satisfaction, quality of health care, ultrasonography

## Abstract

**Background:**

Point-of-care ultrasound is a valuable bedside tool that, with adequate training, can reduce diagnostic uncertainty and improve clinical accuracy. Abdominal pain is a frequent complaint in emergency departments and often requires imaging for appropriate management.

**Objective:**

This study aims to assess the impact of bedside clinical ultrasound performed by family physicians on length of stay, number of basic radiological tests, and need for further diagnostic evaluations in adult patients with abdominal pain.

**Methods:**

This is a pilot quasi-experimental study assessing feasibility and viability, with a nonrandomized control group, to be conducted in the Emergency Department of Hospital Comarcal de Riotinto. Adult patients (≥18 y) presenting with abdominal pain will be included. Both groups will receive standard care. In the intervention group, bedside ultrasound will be performed by trained family physicians; in the control group, ultrasound will be performed by radiologists only if deemed necessary. The primary outcome is the improvement in quality of care, assessed through a reduction in emergency department length of stay, fewer basic radiology tests requested, and diagnostic concordance. Secondary outcomes include the need for additional diagnostic studies and the appropriateness of referrals, evaluated through 1-month follow-up and reconsultation.

**Results:**

The first phase of the project began in 2023 with the validation of the data collection form. Subsequently, the patient satisfaction questionnaire was validated, and the results were published in the journal *Care Primary*. The study has received external funding, and patient recruitment is currently ongoing and expected to be completed in December 2025.

**Conclusions:**

This study aims to demonstrate the clinical and organizational benefits of implementing bedside ultrasound by family physicians in emergency care.

## Introduction

Ultrasound is a diagnostic technique based on the emission and reception of high-frequency waves, so-called ultrasound. It is a patient-friendly technique but requires an interpretation of possible artifacts, as well as progression through a learning curve. Ultrasound in the clinical setting complements physical examination in the same medical procedure, as it is accessible and fast and can be reproduced [1].

The use of ultrasound in emergencies began between 1991 and 1992 by surgeons in the United States, and later in 1996, the acronym FAST (Focused Abdominal Sonography for Trauma) was adopted [2]. From then on, the term POCUS (point-of-care ultrasound) also became commonly used [3].

A review in 2019 showed that POCUS was used for a variety of conditions, although most focused on abdominal and obstetric indications with a good level of diagnostic accuracy [4]. Therefore, it seems appropriate to focus this study on abdominal pain, a common pathology encountered in the emergency department. In fact, a 2010 review indicated that the percentage of nonspecific pain was 24%‐44%, followed by acute appendicitis (15.9%‐28.1%), acute biliary disease (2.9%‐9.7%), and bowel obstruction or diverticulitis in older adults [5].

The pathology related to abdominal pain is highly varied, and training in clinical ultrasound requires a minimal learning curve. Consequently, a number of studies have assessed the training and education of professionals in the detection of biliary tract−related diseases. In this study [6], an acceptable level of agreement was found in the detection of hepatobiliary pathology among junior emergency medicine trainee doctors and radiologists after a short training program. In fact, ultrasound performed by emergency department clinicians affects the diagnostic certainty of abdominal pain in the right hypochondrium, particularly when requesting additional diagnostic studies or in patients with uncertain pathology [7]. The ability of emergency department physicians to diagnose cholelithiasis with bedside ultrasound has also been evaluated [8] and more recently also to rule out cholecystitis [9]. Furthermore, a recent review supports the use of POCUS for gallbladder examination by emergency physicians [10], and another review also suggests that this scan may help in arranging outpatient follow-ups if symptoms have disappeared [11].

In the diagnosis of nephrourological pathology such as renal colic, clinical ultrasound is a good diagnostic tool with a high level of accuracy in detecting urinary tract dilation [12]. These patients may also benefit from an improved level of risk stratification [13]. An observational study has already validated an algorithm for patients with flank pain in the examination and detection of urolithiasis [14]. The use of computed tomography for this pathology has increased, so a review was carried out to compare ultrasound as an alternative and safe test for the patient, which concluded that there is sufficient evidence to determine a more rational algorithm for the management of renal colic [15]. The use of POCUS was also found to be associated with a shorter emergency department stay compared to computed tomography [16].

In the emergency department, the length of stay is determined by many factors, such as the patient’s age, the need for laboratory tests, or referral for consultation, but another factor to be taken into account is the need for ultrasound in abdominal pain [17]. In view of the limited time in the emergency department, ultrasound examination by family doctors is regarded as helping to improve the overall diagnosis and treatment of patients [18]. A recent study from October 2022 also analyzed the effect of POCUS in the initial assessment of patients with acute abdominal pain by evaluating the diagnosis and the decrease in patient waiting time without significant changes in the cost of the service [19].

Many of these patients presenting with abdominal pain at the emergency department require the performance of various complementary tests such as simple radiography. However, the diagnostic efficiency of abdominal radiography in urgent pathologies is not related to the high number of examinations performed as reported in this publication [20]. It is sometimes performed as a routine test despite recommendations to reduce radiation doses to patients and to select pathologies requiring radiography more carefully. This review has already indicated that the use of plain radiography in abdominal pain should be limited to very specific indications [21]. The overuse of these complementary tests leads to unnecessary exposure and increased waiting times in the emergency department. This has been supported by another systematic review [22] where 38 original studies were collected, and the conclusion was that, in most cases, their indiscriminate routine use is not indicated.

Finally, the assessment of the quality of health care is complex and highly subjective as it depends on both the care itself and the patient’s expectations. This analysis of the patient’s experience was carried out using a patient questionnaire, in which the patient’s experience of receiving ultrasound scanning at the point of care was rated positively [23]. However, among the multidimensional models for the assessment of the quality of services provided are the SERVQUAL and SERVPERV questionnaires. The SERVPERF model has been used to assess the perception of quality of care in an emergency department [24], outpatient departments [25], hospital units [26], primary care [27], and even reassessment during the COVID-19 pandemic [28]. The score is calculated by the sum of the scores, and the quality of service is deemed to be as high as the total of the sum of the scores [29]. The SERVPERF model allows the level of quality to be measured by patient assessment.

Accordingly, considering that POCUS can assist professionals in the emergency department to exclude serious causes of abdominal pain−related pathologies, as well as to detect other pathologies that could be followed up at home, this study is proposed to assess the impact of its implementation in a hospital emergency department. Furthermore, the implementation of clinical ultrasound is intended to improve the quality of care and analyze the degree of patient satisfaction.

## Methods

### Study Design and Setting

A quasi-experimental pilot study of feasibility and viability with a control group will be carried out at Hospital Riotinto’s Emergency Department. This center is located in the north of Huelva (Spain) and serves a population of almost 70,000 inhabitants with a high geographical distribution, as some localities are more than 80 km away from the hospital.

This study does not require registration in ClinicalTrials.gov as it does not meet the criteria for a clinical trial according to the International Committee of Medical Journal Editors and US Food and Drug Administration definitions. The project is a quasi-experimental feasibility and implementation study conducted in a real-world clinical setting, with no randomization, no experimental interventions, and no administration of investigational drugs or devices. The study aims to evaluate the impact and utility of point-of-care ultrasound performed by family physicians in emergency departments as part of standard clinical practice. Therefore, it is considered a health services research study rather than a clinical trial, and formal registration is not applicable.

The implementation of bedside clinical ultrasound performed by family physicians in the emergency department is hypothesized to reduce patient length of stay as well as the need for additional diagnostic tests, such as plain radiography or specialist-performed ultrasound, thereby improving the overall quality of patient care. The primary objective is to analyze the impact of this intervention on length of stay, the number of simple radiology tests requested, and the necessity for further examinations by radiodiagnostic specialists.

Specific objectives include determining the length of stay for patients receiving clinical ultrasound by family physicians compared to usual care, quantifying and comparing the number of radiographs and ultrasounds performed, and analyzing patients’ diagnostic and clinical progression over at least 1 month of follow-up. In cases where the process is not resolved, the patient can be monitored for the next 6 months. Additionally, the study aims to evaluate the number and outcomes of specialist-requested ultrasounds in relation to those performed by family physicians, identify factors influencing care quality related to patient health and sociodemographic characteristics, perform diagnostic concordance analyses, and assess patient satisfaction with the service provided.

The sample will consist of patients over the age of 18 years presenting with abdominal pain in the emergency department, who will be assigned to the experimental group (POCUS is performed) or the control group (no clinical ultrasound is performed) consecutively. Patients readmitted for the same pathology will be ruled out. Patients will be invited by the investigators to participate freely, and the recruitment will end when the minimum sample size is reached in each cohort.

The inclusion and exclusion criteria are presented in Textbox 1.

Textbox 1. **The inclusion and exclusion criteria.**

**Inclusion criteria**

- Patients treated at the Emergency Department of Riotinto Hospital during the study and follow-up period
- Patients presenting with acute de novo abdominal pain, defined as a clinical condition causing pain localized by the patient between the thorax and the pelvis. Additionally, patients must not have been admitted for the same condition in the previous 3 months.
- Age ≥18 years
- Patients with sufficient capacity to provide written informed consent
- Experimental group: Patients will be attended by physicians from the Emergency Department of Riotinto Hospital and by professionals with demonstrated training in clinical ultrasound
- Control group: Patients will be attended by physicians from the Emergency Department of Riotinto Hospital and by professionals without demonstrated training in clinical ultrasound

**Exclusion criteria**

- Patients with readmission due to abdominal pain
- Patients who leave the emergency department before completing care
- Patients followed in other regional health systems or private health care
- Pregnant women
- Patients with morbid obesity
- Patients with severe mental illness
- Patients whose clinical severity prevents obtaining informed consent

- Intercurrent illness that makes participation impossible

The reasons for study discontinuation are as follows: (1) patient withdrawal, (2) withdrawal of informed consent, (3) death, (4) protocol violation, and (5) intercurrent illness preventing continued participation.

Informed consent will be obtained by the attending physician at the time of patient evaluation, prior to any study-related procedures.

For the sample size calculation, emergency department length of stay was used as the primary variable, as it is the only parameter that has been studied to date (19). A 25% reduction in the mean length of stay is expected in the intervention group compared with the control group. According to the multicenter trial [30], the mean length of stay for patients with nontraumatic abdominal pain in the emergency department was 5.2 (SD 3) hours. Assuming a 25% reduction (1.3 h) in the intervention group, with a 2-sided significance level of 0.05 and a power of 80%, and applying a Student *t*-test for independent samples, a minimum of 84 patients per group is required. Considering an anticipated 15% attrition rate, the total number of participants to be recruited will be 194 patients (97 per group).

There will be no randomization of the sample, as the 2 groups will receive the same care as normal for their pathology; however, in the experimental group, the intervention (clinical ultrasound) will also be carried out to test its effectiveness and assess whether it improves the quality of care. It is important to consider that clinical ultrasound is not a regulated ultrasound, but rather a response to a clinical question; therefore, it is not a complex intervention, and with the usual pressure of the service, it can be performed at any time of the year.

To achieve the target sample size, recruitment will be conducted consecutively among eligible patients presenting with abdominal pain at the Emergency Department. All family physicians participating in the study have been informed and trained to identify and invite potential participants. The collaboration of the entire emergency care team will be essential to ensure continuous enrollment during the study period. Regular follow-up meetings will be held to monitor recruitment progress and address potential barriers.

Prior to this, training will be provided to professionals, and a common understanding will be reached on the possible main findings. This is intended to reduce clinical variability and improve the external validity of the study.

Given the limited sample size, short duration, and minimal risk involved in this pilot quasi-experimental study, a formal independent data monitoring committee has not been established. The principal investigator and the co-investigators will be responsible for monitoring trial conduct, data accuracy, and participant safety. As no data monitoring committee is planned, further details regarding its charter are not applicable.

No interim analyses are planned for this study due to its exploratory nature and short timeframe. Consequently, no predefined stopping rules have been established. The final decision to complete or terminate the study will rest with the principal investigator, based on recruitment feasibility, protocol adherence, or unforeseen safety concerns.

To ensure data integrity, a quality control procedure has been designed, including predefined field validation rules within the case report form. Participating clinicians were trained during project initiation to minimize data entry errors. Data entry will follow the case report form sequence, and filters will prompt the confirmation of extreme or illogical values. The principal investigator will perform monthly audits, and a second investigator will periodically review the database or a sample of its records. Questionnaire variables will be coded numerically, and preliminary analyses will be performed to detect inconsistencies or missing data. All efforts will focus on minimizing missing data and correcting errors promptly to reduce potential biases and ensure the reliability of the findings.

All individual participant data will be deidentified and securely stored in the REDCap platform hosted by semFYC. Access to the data dictionary, statistical analysis code, and related materials will be available upon reasonable request to the corresponding author, in accordance with ethical and data protection regulations. These materials will be used exclusively for academic or research purposes.

Table 1 shows the summary with the most frequent coded findings in clinical ultrasound.

**Table 1.** Summary with the most frequent coded findings in clinical ultrasounds.

	Encoding	Image
Renal ultrasound		
Kidney cyst	E1	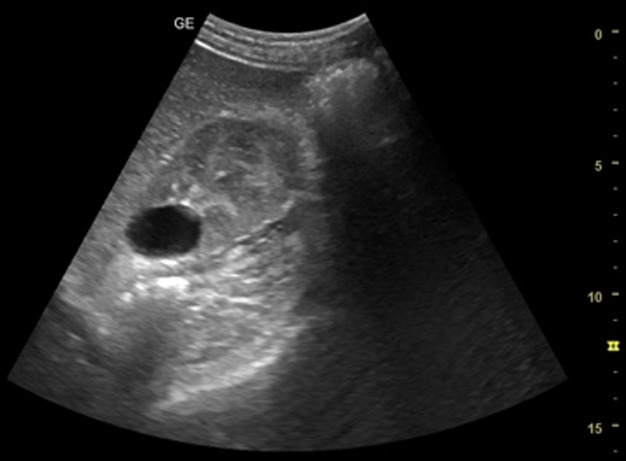
Renal pelvis dilatation	E2	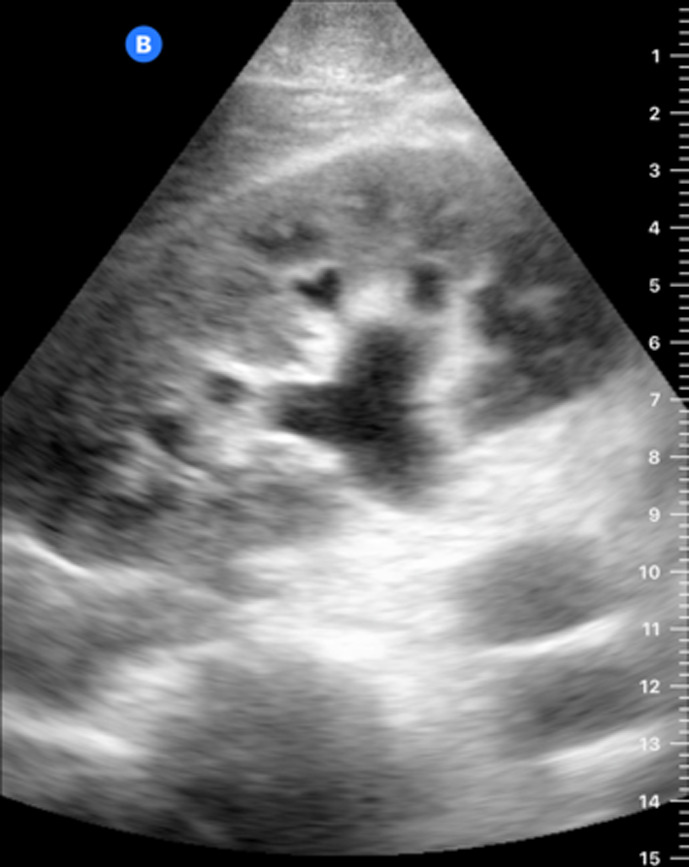
Bladder polyp	E3	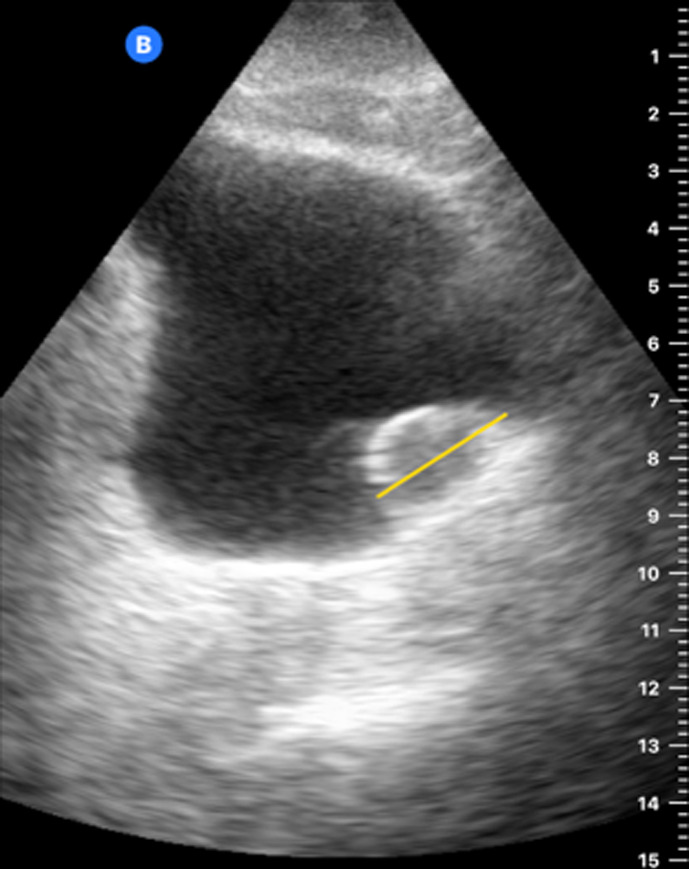
Kidney stones	E4	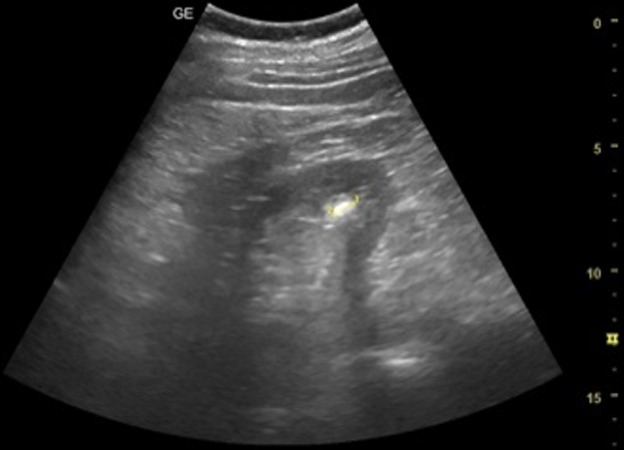
Other (specify)	E5	
Hepatobiliary ultrasound		
Cholelithiasis	H1	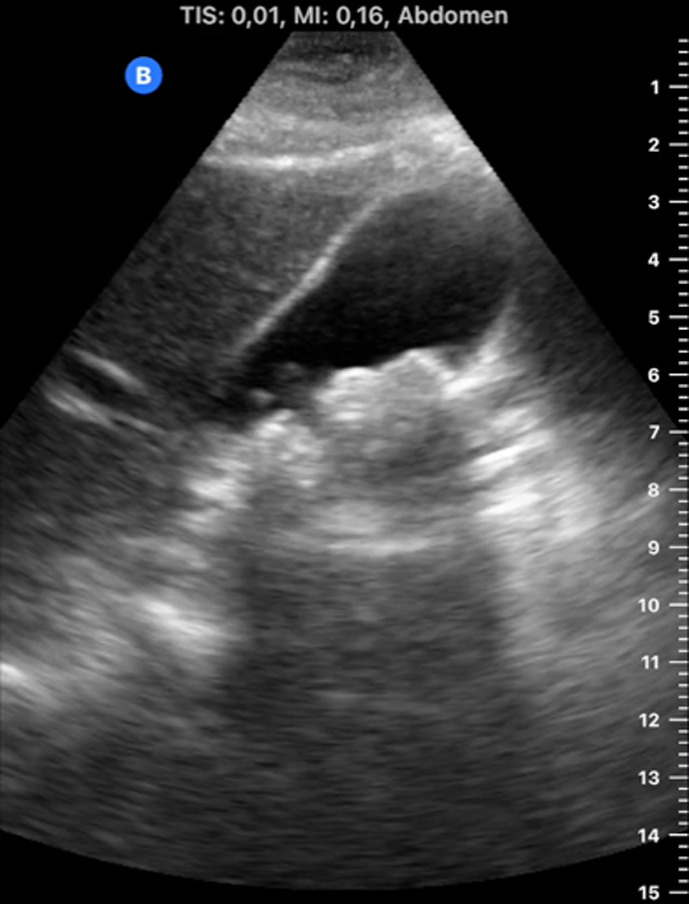
Biliary sludge	H2	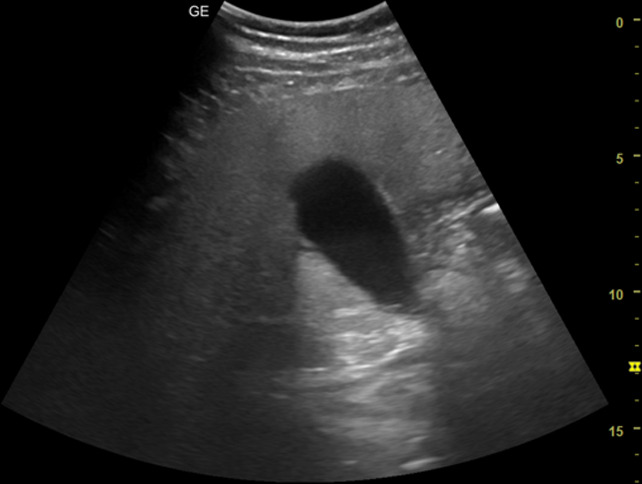
Cholecystitis	H3	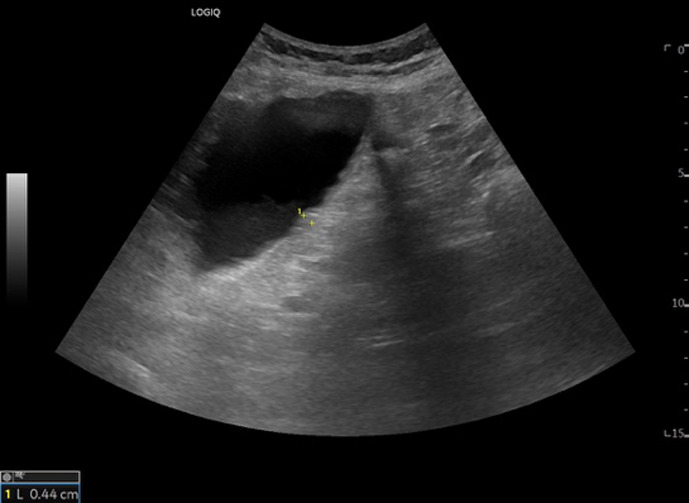
Liver angioma	H4	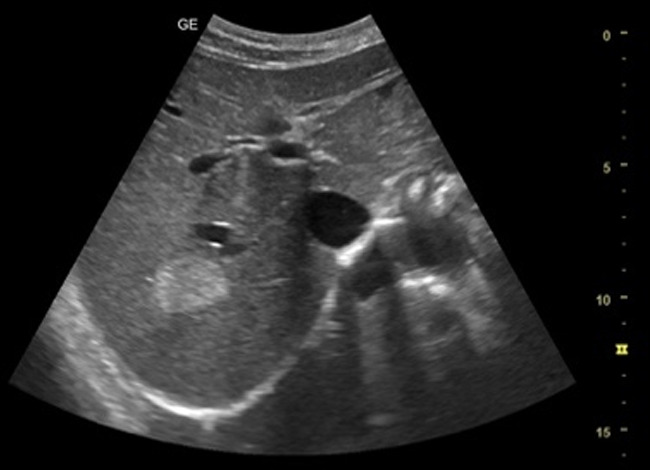
Metastasis	H5	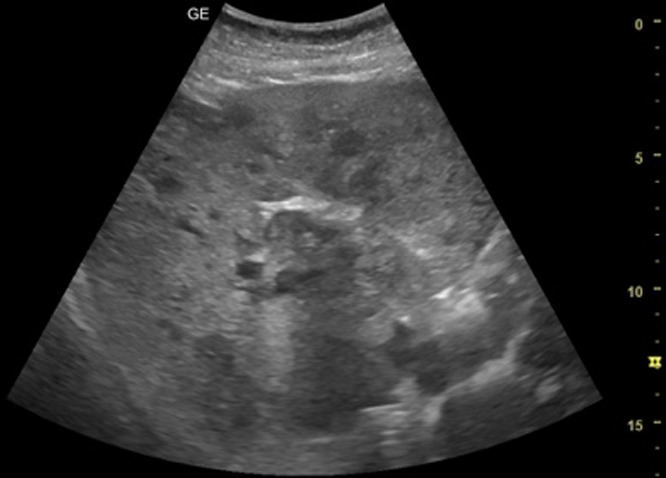
Liver cyst	H6	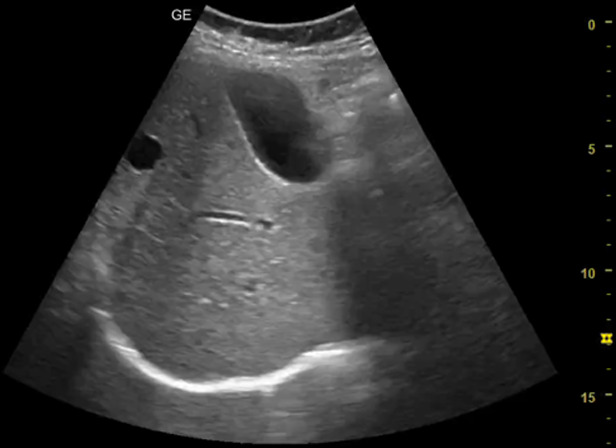
Other (specify)	H7	
Abdominal trauma		
Abdominal free liquid	T1	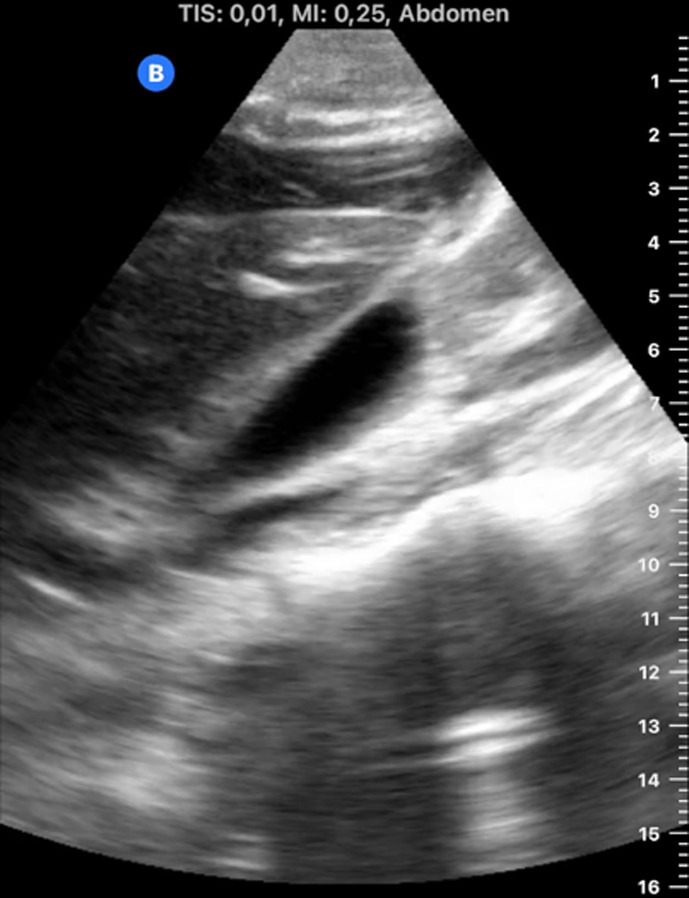
Other (specify)	T2	

**Figure 1.**
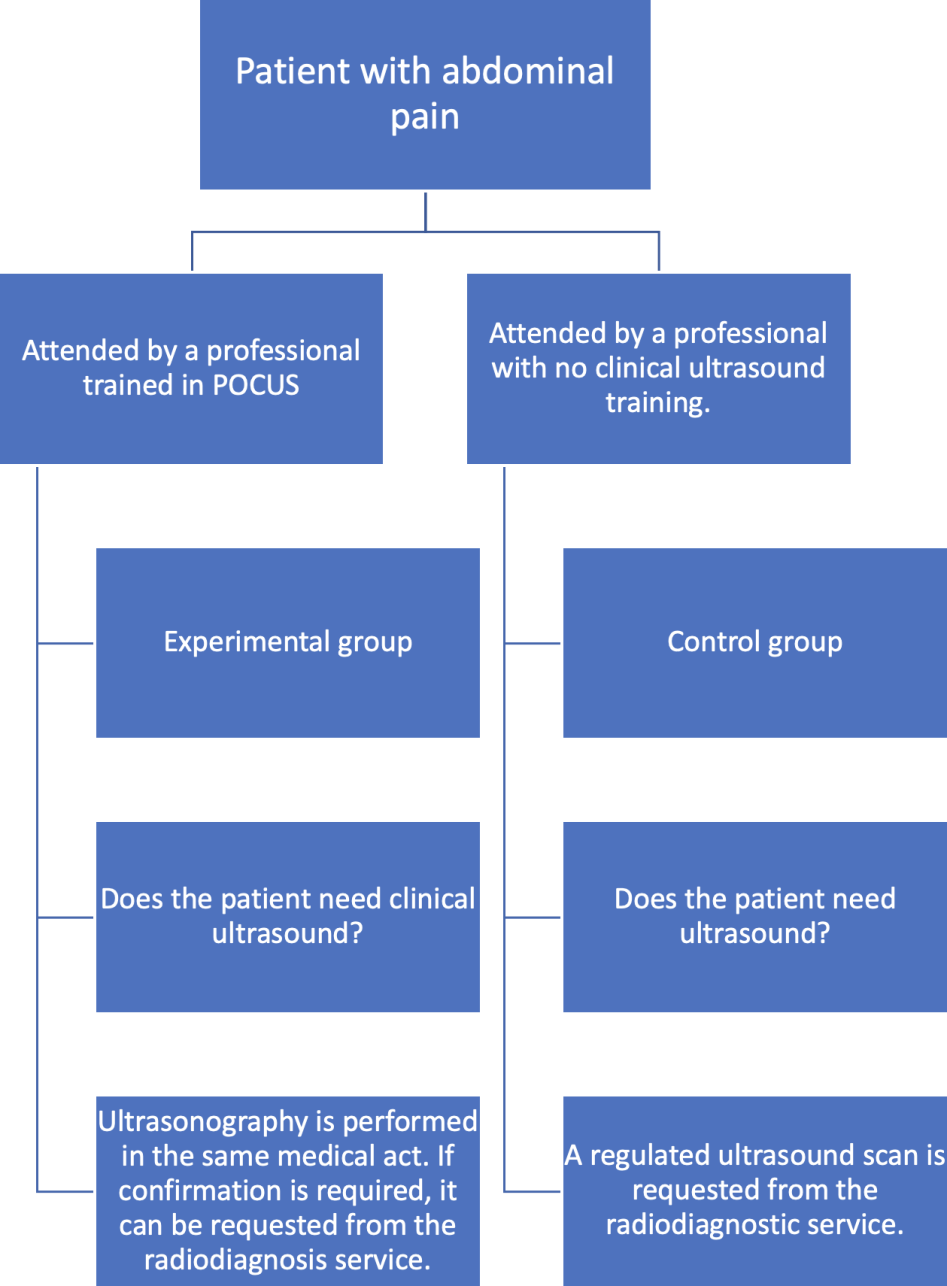
Flowchart depicting study protocol. POCUS: point-of-care ultrasound.

The study protocol is represented in Figure 1.

Subjects will be observed for a period of 1 month to determine whether they have returned for a repeat consultation for the same reason, and if so, to check the degree of alignment of the final diagnosis with the initial care. Only in cases where the process is not resolved can the patient be monitored for the next 6 months.

### Statistical Analysis

A descriptive analysis will be carried out including proportions, measures of central tendency, and measures of dispersion, according to the nature of the variables (quantitative variables expressed as mean and SD and qualitative variables as a percentage). The analysis will include description and statistical analysis to identify any significant differences between men and women.

Comparison of the variables of interest and the stratification and potentially confounding variables at the start of the study will be carried out in both groups. The baseline values of the study variables will be tested for their homogeneity between the two groups (Student *t*-tests and chi-square tests, depending on the nature of the variables). There will also be a normality check for variables such as length of stay and those that require it.

The emergency department length of stay will be compared using a Student *t* test for independent samples, and a multiple linear regression model will be applied to adjust for potential confounding factors. The number of plain radiographs and complementary imaging studies in the Radiology Department will be analyzed using Poisson or logistic regression models, as appropriate. To account for multiple hypothesis testing, a Holm-Bonferroni correction will be applied.

The possible existence of potential confounding factors or the interaction of other variables in the relationship between the proposed intervention and the outcome variables will be assessed using logistic regression models. For continuous variables, it will be assessed by multiple linear regression.

In all cases, a statistical significance of 5% (*P*<.05) is required. The data will be processed and analyzed with SPSS (version 25.0; IBM Corp).

### Variables

The main outcome variables aligned with the study objectives include the length of stay in the emergency department (measured in minutes), the number and type of radiological tests requested, the performance of bedside ultrasound (POCUS), diagnostic concordance, and the patient’s satisfaction with the care received. Sociodemographic variables such as age, sex, and distance from home, as well as clinical and procedural variables, will also be collected.

A complete list of all study variables, including their measurement units or response categories, is provided in Table 2, grouped into thematic blocks: sociodemographic, clinical, diagnostic, and outcome variables.

**Table 2.** Study protocol variables.

Block and variable		Unit or format	Notes or response options
Sociodemographic			
	Identification code	Alphanumeric	Unique ID per patient
	Gender	Categorical	Male or female or other
	Date of birth	Date (dd/mm/yyyy)	Age will be calculated
	Distance to home	Kilometers	Estimated by postal code
Clinical			
	Health problems	Free text or ICD-10^a^	Comorbidities
	Current medications	Free text	List of active treatments
	Vital signs	Numeric (per parameter)	BP^b^ (mm Hg), HR^c^ (bpm), EVA^d^, temperature (°C), SatO_2_ (%)
	Reason for consultation	Free text	Chief complaint
Care process			
	Attending doctor	Code or initials	Family physician ID
	Date or time of arrival	Time stamp	Format: dd/mm/yyyy hh:mm
	Date or time of discharge	Time stamp	Format: dd/mm/yyyy hh:mm
	Origin	Categorical	Self-referred or primary care or ambulance or other
	POCUS^e^ performed	Binary	Yes or no
	POCUS findings	Free text	Based on clinical protocol
	Other tests requested	Categorical or multiple	Blood tests or ECG^f^ or other
	Radiology test performed	Categorical	X-ray or formal ultrasound or CT^g^ or none
	Interconsultation	Binary	Yes or no; specialty if applicable
Outcome			
	Diagnosis	Free text or ICD-10	Final ED^h^ diagnosis
	Discharge referral	Categorical	Home or hospitalization or specialist or follow-up
	Length of stay	Minutes	Calculated from entry and discharge times
	Satisfaction questionnaire	Likert scale or categorical	1‐5 scale or qualitative responses
	Diagnostic concordance	Binary	Yes or no (based on follow-up or second opinion)

a *ICD-10: International Classification of Disease, Tenth Revision.*

b BP: blood pressure.

c HR: heart rate.

d EVA: Escala Visual Analógica (VAS: visual analog scale).

e POCUS: point-of-care ultrasound.

f ECG: electrocardiogram.

g CT: computed tomography.

h ED: emergency department.

Table 3 shows the results of the satisfaction questionnaire based on the SERVPERF model.

**Table 3.** Satisfaction questionnaire based on the SERVPERF model^a^.

Dimensions and questions		Score range (strongly disagree to strongly agree)
Tangible elements		
	The facilities are suitable for patient care	1-7
	Technological equipment is adequate	1-7
	The emergency department has sufficient capacity to attend to the population	1-7
Responsiveness dimension		
	If you have any questions, you will receive a response within a reasonable time	1-7
	The attention of the staff provides a fast and high-quality service	1-7
	You have been dealt with quickly and promptly	1-7
Reliability dimension		
	When staff commit to doing something within a certain time frame, it is fulfilled	1-7
	If you have a problem, the staff show an interest in solving it	1-7
	Staff perform the service well the first time	1-7
	Professionals are ready to help when they are needed	1-7
Security dimension		
	Staff behavior imparts confidence	1-7
	There is a capacity to resolve doubts accurately	1-7
	Staff are knowledgeable when responding to queries	1-7
Empathy dimension		
	Care is offered that responds to the needs of the users	1-7
	Staff care about your interests and needs	1-7
	The emergency service provides a solution to the health needs of users	1-7
Access dimension		
	The waiting time for service was adequate	1-7
	Waiting time for tests and results was adequate	1-7
	The overall waiting time was adequate	1-7

a Likert scale (1-7) from least to most satisfaction.

Table 4 shows the work schedule for the study protocol.

Potential harms will be defined as any adverse event or unintended consequence resulting from the use of bedside ultrasound by nonradiologist physicians. Harms will be assessed systematically, using a structured reporting form completed by the attending physician and monitored through follow-up at 1 month. Any serious adverse events will be documented and reviewed by the principal investigator and the ethics committee if needed.

**Table 4.** Timeline of the study.

		Year 1												Year 2											
	Phase 0	1	2	3	4	5	6	7	8	9	10	11	12	1	2	3	4	5	6	7	8	9	10	11	12
Ultrasound training	✓																								
Literature review update		✓																							
Presentation of the research project in the emergency department			✓																						
Protocol development				✓	✓																				
Design of the data collection form					✓	✓																			
Training and consensus on key pathologies							✓	✓																	
Data collection									✓	✓	✓	✓	✓	✓											
Data cleaning, analysis, and results generation															✓	✓	✓	✓	✓						
Results dissemination and manuscript preparation																				✓	✓	✓	✓	✓	✓

### Ethical Considerations

This study has been approved by the Huelva Provincial Independent Ethics Committee (reference code PEIBA 1923-N-21). Any significant modifications to the protocol will be submitted for approval to the same ethics committee and promptly communicated to all relevant stakeholders, including participating investigators and, where necessary, trial registries.

Informed consent will be obtained by the attending family physicians or authorized research team members prior to the inclusion of any participant. Participants will receive both oral and written information about the study objectives, procedures, risks, and benefits and will be given the opportunity to ask questions before providing written consent. No biological specimens will be collected, and no ancillary studies requiring additional consent are planned. Participants will not receive any financial compensation or incentives for their participation in the study.

Personal data will be collected and stored using anonymized codes to ensure confidentiality. Access to identifiable information will be restricted to the research team and stored securely in password-protected systems in accordance with national data protection regulations. Data will be treated with strict confidentiality before, during, and after the trial, and only aggregated, deidentified data will be reported in publications or presentations.

Given the minimal-risk nature of this study, no specific post-trial care provisions are foreseen. However, if any harm were to occur as a result of participation, appropriate medical care and any necessary support will be provided by the institution in accordance with applicable regulations and institutional policies. The application procedures and the study were approved by the Huelva Provincial Ethics Committee (Comité de Ética de Investigación de Huelva) (Code PEIBA 1923-M1-21).

## Results

The first phase of the project began in 2023 with the validation of the data collection form. Subsequently, the patient satisfaction questionnaire was validated, and the results were published in the journal Care Primary. The study has received external funding, and patient recruitment is currently ongoing and expected to be completed in December 2025.

The project was initiated in 2023. Between March and July 2023, an initial validation phase of the data collection form was conducted. Preliminary findings were presented as a clinical communication at the 44th semFYC National Congress, held in Barcelona, 14‐16 November 2024. A total of 65 patients were included in this first sample, with a mean age of 56.6 (SD 20.0) years, of whom 53.8% (n=35) were women and 46.2% (n=30) were men. The validation of the data collection form showed that 76.9% (n=50) of the ultrasound examinations were performed as point-of-care clinical ultrasounds (POCUS), whereas only 6.2% (n=4) were formal imaging studies referred to the Radiology Department.

Based on this pilot experience, the study protocol was further developed. Funding was subsequently obtained through the 2024 Call of the Fundación Progreso y Salud (Andalusian Public Health System) under the Research and Innovation Projects category in Primary Care, Regional Hospitals, and High-Resolution Healthcare Centers (grant ID: AP-0561‐2024-C5-F2).

Further work focused on refining the study protocol and validating the patient satisfaction questionnaire was published in [31].

Patient recruitment is currently ongoing and is expected to be completed by December 2025. We anticipate that the implementation of POCUS in patients presenting with abdominal pain in emergency settings will lead to a reduction in length of stay and a decrease in the use of additional diagnostic tests, such as plain radiography or formal radiology−performed ultrasound.

## Discussion

### Principal Findings

POCUS has emerged as a valuable tool in emergency medicine, particularly for its potential to reduce diagnostic uncertainty and enhance clinical accuracy in decision-making. Despite these recognized advantages, there is limited evidence regarding its overall impact on the quality of care, and even less from the patient’s perspective. This study protocol is based on the hypothesis that the implementation of clinical ultrasound performed by family physicians in emergency departments can reduce length of stay and decrease the need for complementary diagnostic tests, such as plain radiography or radiology-performed ultrasound. Collectively, these improvements may lead to a measurable enhancement in both perceived and actual quality of care.

The geographic context of this study adds a relevant dimension to the analysis. The target population resides in an area characterized by wide territorial dispersion and significant population aging, factors that place additional demands on emergency services. In such settings, clinical practice often prioritizes the resolution of health problems in a single visit to avoid repeated travel—especially for patients with reduced mobility or limited access to transportation. POCUS performed by emergency physicians may therefore not only optimize diagnostic workflows but also align more closely with the health care needs of the community.

One of the main methodological challenges in this study is interoperator variability in the interpretation of ultrasound findings. To minimize this, a reference image repository has been developed, alongside simplified visual guides to standardize the use of clinical ultrasound in patients presenting with abdominal pain. In addition, prestudy training sessions were conducted in collaboration with the radiology department to promote consistency in image acquisition and interpretation among participating physicians.

Beyond evaluating diagnostic efficiency, the study also aims to identify the key factors contributing to improved quality of care from the health care professional’s perspective. This approach will provide both qualitative and quantitative insights into the clinical, organizational, and human elements that influence better health outcomes.

Finally, this research initiative may pave the way for broader applications of clinical ultrasound beyond hospital-based emergency settings, including prehospital care and primary health care services. If the results are favorable, they could support the development and implementation of standardized protocols for the use of POCUS by family physicians, with potential implications for health care system efficiency and patient-centered outcomes.

### Conclusion

This study aims to assess whether the implementation of POCUS performed by family doctors in the emergency department reduces the length of stay in the department, as well as other diagnostic tests such as simple radiology or regulated ultrasound, improving the quality of patient care.

## Supplementary material

10.2196/82393 Checklist 1 SPIRIT checklist.

10.2196/82393 Peer Review Report 1 Peer review report by the 2024 Call for R&I Projects in Primary Care, Regional Hospitals, and CHARES Review Committee, Fundación Andaluza Beturia para la Investigación en Salud (FABIS; Beturia Andalusian Foundation for Health Research, Spain.

## Abbreviations

POCUS: point-of-care ultrasound
