# Supplementary material for: Bedside Clinical Ultrasound Performed by Family Physicians in Adult Patients With Abdominal Pain in a Hospital Emergency Department: Protocol for a Pilot Quasi-Experimental Study
Source: JMIR Res Protoc. 2026 Jan 2;15:e82393. doi: 10.2196/82393 (PMC12758840; doi:10.2196/82393)
Supplement: Peer Review Report 1 [file resprot-v15-e82393-s002.pdf]

INFORME FINAL DE EVALUACIÓN
Convocatoria 2024 de Proyectos de I+i en atención primaria, hospitales comarcales y CHARES.

**Modalidad**	Proyectos de Investigación en AP.
**Expediente**	AP-0561-2024-C5-F2
**Titulo del proyecto**	Impacto del uso de la ecografía clínica en el punto de urgencias realizada por médicos de familia en el dolor abdominal
**Invest. Principales**	LAURA CARBAJO MARTÍN
**Invest. Colaboradores/as**	Ignacio Párraga Martínez, Máximo Bernabeu Wittel, Esperanza Macarena Ortega Hidalgo, Juan Jesús Rosado Cabral, Jacqueline Trueba Carreón, ANA MARIA BENJUMEDA GONZALEZ, María José Rosende Domínguez, Noelia Trujillo Díaz
**Entidad beneficiaria**	Fundacion Andaluza Beturia para la Investigacion en Salud
**Centro/s**	H. Virgen del Rocío, H. Riotinto (A.G.S. Norte de Huelva), FABIS-Fundación Andaluza Beturia para la Investigación en Salud, Gerencia de Atencion Primaria de Albacete
**Puntuación del proyecto**	**82.81**

Una vez finalizada la fase de evaluación, siguiendo los criterios recogidos en el apartado sexto de la convocatoria de proyectos de Investigación e innovación en el ámbito de la atención primaria y hospitales comarcales y centros hospitalarios de alta resolución del Sistema Sanitario Público de Andalucía, **los resultados de la evaluación del proyecto** indicado anteriormente son los siguientes:

	**Criterios de Evaluación**	**Puntuación del Proyecto**	**Puntuación máxima posible**
**49_00 Calidad científico-técnica del proyecto**	80_00 Hipótesis y objetivos	8,50	10
	81_00 Grado de conocimiento sobre el tema a abordar	9	10
	82_00 Viabilidad y riesgos o limitaciones de la propuesta	13	15
	83_00 Metodología y plan de trabajo	13,50	15
	**49_00 Calidad científico-técnica del proyecto**	**44**	**50**
**50_00 Valoración del equipo investigador/innovador**	44_00 Dedicación y composición del equipo	4,50	5
	84_00 Capacitación científico técnica de la/s persona/s que lidera/n el proyecto	8	10
	45_00 Capacitación científico técnica del equipo	7,50	10
	**50_00 Valoración del equipo investigador/innovador**	**20**	**25**
**52_00 Adecuación del presupuesto**	85_00 Valoración del presupuesto	5	5
	**52_00 Adecuación del presupuesto**	**5**	**5**
**Criterios estratégicos**	Criterios estratégicos	2	5
	14_00 IP Mujer	2	2
	284_00 Perspectiva de género en la propia investigación	0	3
	**Criterios estratégicos**	**2**	**5**
**Aplicabilidad**	**Aplicabilidad**	**11,81**	**15**
	**Puntuación total del proyecto**	**82.81**	**100**

(*) *Para que un proyecto de investigación pueda ser financiado deberá obtener, al menos, 25 puntos en el criterio 49_00 Calidad científico-técnica del proyecto, al menos 7,5 puntos en el criterio de Aplicabilidad y 60 puntos en la suma de todos los criterios.*

1. COMENTARIOS ACERCA DE LA EVALUACIÓN

80_00 Hipótesis y objetivos (8.5/10.00)

La temática del estudio es de relevancia clínica.

La hipótesis se ajusta a la pregunta de investigación del estudio, y está claramente definida, pero requeriría cuantificación.

El objetivo principal es claro y está bien redactado.

Los objetivos específicos han mejorado en su redacción respecto a la versión de la convocatoria previa.

El concepto calidad de la asistencia no está suficientemente clarificado.

81_00 Grado de conocimiento sobre el tema a abordar (9.0/10.00)

Bibliografía adecuada a la temática del estudio, actualizada y ajustada a normas de Vancouver. Del total de 38 citas bibliográficas 20 (52,6%) corresponden a los últimos 5 años (2019-2024) y 2 del año en curso (2024).

El apartado elaborado a partir de esta bibliografía tiene un contenido adecuado al objetivo del estudio.

Se sigue recomendando revisar el orden de los contenidos, para mejorar el hilo argumental del apartado.

82_00 Viabilidad y riesgos o limitaciones de la propuesta (13.0/15.00)

Este es uno de los aspectos más importantes del proyecto:

Varios aspectos han sido clarificados respecto a la propuesta previa, como la capacitación de los profesionales que formarán los grupos intervención y control, así como los pacientes asignados a los mismos.

También se ofrece mayor información sobre las características de la formación recibida por los médicos de familia que se incluirán en le grupo intervención. Esta nueva información permite apreciar una mayor vialidad de la propuesta y una mayor relevancia de los potenciales resultados. No obstante, la propuesta se beneficiaría de una mayor clarificación sobre cómo se llevará a cabo la inclusión de pacientes (por ejemplo clarificar la gravedad de los cuadros que se incluirán) y el seguimiento a realizar al mes y a los 6 meses,

83_00 Metodología y plan de trabajo (13.5/15.00)

El diseño planteado es adecuado al contexto y al objetivo principal del estudio.

Algunas de las causas de finalización del estudio podrían ser criterios de exclusión; esto debería ser clarificado.

En el cálculo del tamaño muestral se supone un 25% de diferencias basadas en una bibliografía concreta, pero no se especifica a qué diferencias se refieren.

Las mejoras planteadas en la evaluación previa en cuanto al reclutamiento de pacientes han sido introducidas. Se justifica la necesidad de seguir capacitando al profesional del grupo intervención, por lo que la intervención en si misma es la atención que recibe el paciente con dolor abdominal por parte de un profesional bien capacitado en el manejo de la ecografía clínica que realizará una prueba ecográfica como parte de la orientación diagnóstica del paciente (esto debería ser claramente recogido en la propuesta).

El seguimiento de los pacientes ha sido clarificado, aunque se debería plantear de manera mas detallada que se hará al mes y que se hará a los 6 meses y a quiénes.

Las variables están mejor definidas, con más detalles sobre el procedimiento de medida. Se sugiere especificar de forma más estructurada y clara, la jerarquía de las mismas (en términos de variables dependientes o resultado incluyendo resultados primarios y secundarios, variables independientes y modificadoras de efecto así como las posibles confusoras). En el CRD no se incluye la frecuencia respiratoria, elemento básica para valorar la gravedad de los pacientes y el nivel de dolor en determinados grupos poblacionales.

La utilización de un cuaderno de recogida de datos previamente estandarizado y filtrado en REDCap es un buen mecanismo para garantizar la calidad de los datos, y se ofrecen más detalles sobre el procedimiento de corrección de errores que se plantea por parte de la IP y los circuitos de información del estudio.

Las estrategias de análisis de datos han sido modificadas y se ajustan a los objetivos del estudio; se sugiere trabajar más a fondo la variable sobre la evaluación de la calidad de la atención que se pide valore el paciente mediante el modelo SERVPEF.

Los tiempos recogidos en el cronograma son adecuados a las tareas.

Los aspectos éticos se ajustan a lo requerido para este tipo de estudios, además el trabajo ya cuenta con la aprobación por un comité de ética.

Se sugiere mejorar los contenidos de la hoja de información al paciente (por ejemplo, usar siempre el usted, añadir los grupos del estudio, eliminando términos técnicos...). No se especifican entregables.

44_00 Dedicación y composición del equipo (4.5/5.00)

El equipo que presenta esta propuesta es amplio (9 investigadores) y adecuado al proyecto. Está compuesto por médicos de diferentes especialidades, principalmente medicina de familia (6), medicina interna (1), radiología (1) y técnico de apoyo a la investigación (1).

El reparto de tareas es adecuado.

84_00 Capacitación científico técnica de la/s persona/s que lidera/n el proyecto (8.0/10.00)

La investigadora principal expone en su curriculum una amplia trayectoria profesional e investigadora, especialmente en lo que se refiere a la participación en congresos y jornadas científicas.

Recientemente ha incrementado su producción científica en lo relacionado con las publicaciones (5 artículos en los últimos 5 años, en revistas sin factor de impacto)

45_00 Capacitación científico técnica del equipo (7.5/10.00)

Algún miembro del equipo investigador presenta amplia trayectoria investigadora, junto a otros con actividad moderada, pero una parte importante del mismo describen una trayectoria investigadora limitada.

85_00 Valoración del presupuesto (5.0/5.00)

Presupuesto adecuado al proyecto.

Criterios estratégicos (2.0/5.00)

14_00 IP Mujer (2.0/2.00)

El 100% de las IPs son mujeres

284_00 Perspectiva de género en la propia investigación (0.0/3.00)

No se ha otorgado una valoración positiva a la INCORPORACIÓN DE LA PERSPECTIVA DE GÉNERO EN LA INVESTIGACIÓN, al considerar que no contempla los criterios mínimos establecidos por la Comisión de evaluación de la perspectiva de género en la convocatoria.
Se detallan a continuación los criterios valorados:

- C1 (Criterio relacionado con la formulación del problema): Se define el problema de investigación teniendo en cuenta las diferencias y similitudes entre mujeres y hombres (en la formulación del problema de estudio, objetivos, hipótesis?).
- C2 (Criterio relacionado con la recogida de datos):¬¬¬ Se plantea una recogida de datos desagregada por sexo o se asegura una adecuada representación de mujeres y hombres en la información que se va a recoger.
- C3 (Criterio relacionado con el análisis de datos): El enfoque analítico es apropiado y suficientemente riguroso como para capturar factores basados en el género o el sexo.
- C4 (Criterio relacionado con la aplicabilidad de los resultados): Se identifican posibles implicaciones de sexo y/o género en los resultados del estudio.
- C5 (Criterio relacionado con el lenguaje): Se utiliza un lenguaje inclusivo y no sexista.

 Si solo se incluye un sexo en el estudio, o si los resultados del estudio se aplican a un sólo sexo, se especifica éste para el caso de humanos, animales o de cualquier célula, tejido u otro material derivado de éstos.
 En cualquier caso, la influencia de los factores relacionados con el sexo y/o el género se determina a priori sobre la base de su papel hipotético en la causalidad, el curso, la eficacia del tratamiento, el impacto y el resultado de los problemas de salud.

Aplicabilidad (11.81/15)

El proyecto tiene un importante alcance y de mostrar resultados positivos contribuirá al fortalecimiento de la realización de esta técnica en por médicos de familia en urgencias hospitalarias. La previsión de implantación depende de modificaciones estructurales en los servicios de urgencias que parece posible.

Comentario Global del Proyecto.

El presente proyecto ha sido modificado respecto al previo, lo que ha generado una propuesta viable, de interés científico y con potencialidad de generar resultados de alcance y transferibles al sistema sanitario, beneficiando la calidad de la atención prestada al paciente con dolor abdominal que acude a urgencias de hospital. Algunos aspectos podrían ser mejorados, principalmente la cuantificación de la hipótesis del estudio, la redacción ordenada del apartado de antecedentes bibliográficos, proporcionar un mayor detalle en los procedimientos de recogida de información al inicio y durante las dos tiempos de seguimiento establecidos. La inclusión de un modelo de evaluación de la calidad de la asistencia es valiosa, pero se debería indicar más detalles sobre como se procederá a su análisis y qué impacto se espera de estos resultados.

2. INFORMACIÓN SOBRE LA INVESTIGACIÓN CONJUNTA CON EMPRESAS Y/O PROTECCIÓN DEL CONOCIMIENTO

La protección del conocimiento generado en los proyectos de I+i constituye un pilar básico en el proceso de valorización y transferencia de resultados de investigación, cuyo objetivo último es garantizar el traslado eficiente y la aplicación de dichos resultados en Salud. Si desea asesoramiento o tiene alguna cuestión relacionada con las colaboraciones con empresas y/o asuntos de propiedad industrial o intelectual, puede ponerse en contacto con los técnicos de la OTT por teléfono (955040450) y correo electrónico (ott.sspa@juntadeandalucia.es).

3. INFORMACIÓN SOBRE OTRAS OPORTUNIDADES DE FINANCIACIÓN Y LOS SERVICIOS QUE PROPORCIONA LA RED DE FUNDACIONES GESTORAS DE INVESTIGACIÓN DEL SSPA (RFGI-SSPA)

Si desea información sobre **otras oportunidades de financiación** tiene a su disposición a través del Portal de Servicios de Gestión y Apoyo a la I+i del Sistema Sanitario Público de Andalucía **INVESTIGA+** las siguientes herramientas:

- Buscador de convocatorias: Encuentra según el tipo de ayuda, ámbito, entidad financiadora, estado o por el nombre, la convocatoria que mejor se ajusta a las necesidades de tu actividad. Acceda

- Agenda de Oportunidades de Financiación para la I+i Biomédica, (formato calendario imprimible en A4). Instrumento de planificación y consulta en el que se incluyen los **requisitos** imprescindibles para la participación en las convocatorias, y un cronograma con los plazos efectivos o previstos para la presentación de solicitudes, y con un **hipervínculo a la ficha detallada** de cada una de las ayudas.
Este año, como novedad, se edita únicamente en formato digital, lo que permite una actualización diaria de las ayudas. Consúltela

Además en **INVESTIGA+** , podrá encontrar toda la información correspondiente a la cartera de servicios que ofrece la Red de Fundaciones Gestoras de la Investigación del Sistema Sanitario Público de Andalucía (RFGI-SSPA). La RFGI-SSPA ofrece apoyo, soporte y personalidad jurídica según su ámbito geográfico. Está compuesta por siete fundaciones: FCÁDIZ (Cádiz), FIBICO (Córdoba), FABIS (Huelva), FIMABIS (Málaga), FISEVI (Sevilla), FIBAO (con carácter interprovincial, dando cobertura a Granada, Jaén y Almería) y la Fundación Pública Andaluza Progreso y Salud (FPS) como entidad coordinadora de la Red.

La **Cartera de Servicios de Apoyo a la I+i** que la RFGI-SSPA ofrece al Investigador/a es la siguiente:

1. Asesoramiento para la captación de financiación

- Difusión de oportunidades de financiación, de búsquedas de socios, de oportunidades de colaboración con empresas (demandas tecnológicas, colaboración público-privada)
- Formación para la planificación de la captación de recursos

2. Gestión de ayudas

- Preparación y seguimiento de las propuestas
- Gestión económica de las ayudas y justificación

3. Gestión de ensayos clínicos y estudios observacionales

- Difusión de oportunidades de investigación clínica y gestión administrativa de ensayos clínicos
- Asesoramiento para la investigación clínica independiente

4. Asesoramiento de Proyectos Internacionales

- Preparación de propuestas y búsquedas de socios
- Formación / Información

5. Apoyo Metodológico y Estadístico

- Formación en búsquedas (BV-SSPA + OTT-SSPA)
- Asesoramiento metodológico (proyectos y tesis doctorales)
- Análisis estadístico y diseño de bases de datos

6. Asesoramiento y gestión de la protección y transferencia de los resultados

- Asesoramiento para las publicaciones y servicios de traducción
- Acuerdos de colaboración público-privada
- Asesoramiento y gestión para la protección y transferencia de resultados
